# Association Between Antimicrobial Stewardship Programs and Antibiotic Use Globally: A Systematic Review and Meta-Analysis

**DOI:** 10.1001/jamanetworkopen.2022.53806

**Published:** 2023-02-09

**Authors:** Kyaw Zay Ya, Phyo Thet Naing Win, Julia Bielicki, Mark Lambiris, Günther Fink

**Affiliations:** 1 Department of Epidemiology and Public Health, Swiss Tropical and Public Health Institute, Allschwil, Switzerland; 2 University of Basel, Basel, Switzerland; 3 Independent researcher, Yangon, Myanmar; 4 Paediatric Infectious Diseases Research Group, Institute of Infection and Immunity, St George’s, University of London, London, United Kingdom; 5 Paediatric Pharmacology and Paediatric Infectious Diseases, University Children’s Hospital Basel, Basel, Switzerland

## Abstract

**Importance:**

Antimicrobial resistance continues to spread rapidly at a global scale. Little evidence exists on the association of antimicrobial stewardship programs (ASPs) with the consumption of antibiotics across health care and income settings.

**Objective:**

To synthesize current evidence regarding the association between antimicrobial stewardship programs and the consumption of antibiotics globally.

**Data Sources:**

PubMed, Web of Science, and Scopus databases were searched from August 1, 2010, to Aug 1, 2020. Additional studies from the bibliography sections of previous systematic reviews were included.

**Study Selection:**

Original studies of the association of ASPs with antimicrobial consumption across health care and income settings. Animal and environmental studies were excluded.

**Data Extraction and Synthesis:**

Following the Preferred Reporting Items in Systematic Reviews and Meta-Analyses guideline, the pooled association of targeted ASPs with antimicrobial consumption was measured using multilevel random-effects models. The Effective Public Health Practice Project quality assessment tool was used to assess study quality.

**Main Outcomes and Measures:**

The main outcome measures were proportion of patients receiving an antibiotic prescription and defined daily doses per 100 patient-days.

**Results:**

Overall, 52 studies (with 1 794 889 participants) measured the association between ASPs and antimicrobial consumption and were included, with 40 studies conducted in high-income countries and 12 in low- and middle-income countries (LMICs). ASPs were associated with a 10% (95% CI, 4%-15%) reduction in antibiotic prescriptions and a 28% reduction in antibiotic consumption (rate ratio, 0.72; 95% CI, 0.56-0.92). ASPs were also associated with a 21% (95% CI, 5%-36%) reduction in antibiotic consumption in pediatric hospitals and a 28% reduction in World Health Organization watch groups antibiotics (rate ratio, 0.72; 95% CI, 0.56-0.92).

**Conclusions and Relevance:**

In this systematic review and meta-analysis, ASPs appeared to be effective in reducing antibiotic consumption in both hospital and nonhospital settings. Impact assessment of ASPs in resource-limited settings remains scarce; further research is needed on how to best achieve reductions in antibiotic use in LMICs.

## Key Points

**Question:**

What is the association between antimicrobial stewardship programs (ASPs) implemented across multiple health care settings and antibiotic use?

**Findings:**

In this systematic review and meta-analysis of 52 studies with more than 1.7 million patients conducted in different health care and income settings, ASPs were associated with reduced consumption of antibiotics overall as well as of antibiotics in the World Health Organization Watch group.

**Meaning:**

The findings of this study support the use of ASPs to reduce antibiotic use in both hospital and nonhospital settings.

## Teaser

This systematic review and meta-analysis synthesizes current evidence regarding the association between antimicrobial stewardship programs and the consumption of antibiotics globally.

## Introduction

Antimicrobial resistance (AMR) continues to spread rapidly at a global scale.^1^ Recent global estimates suggest that the disease burden of AMR is at least as high as that of HIV and malaria combined, with an estimated 4.95 million deaths caused in 2019.^1^ If not properly addressed, AMR could kill 10 million people every year and cost the global economy up to $100 trillion by 2050.^2^

A number of antimicrobial stewardship programs (ASPs) have been introduced in different settings to optimize antimicrobial use and delay resistance, while at the same time ensuring patient safety and avoiding additional health care costs.^3,4,5,6,7,8,9^ The latest research suggests that ASPs can reduce total antibiotic consumption by 19% and the use of restricted antimicrobial drugs by 27% in hospital.^10^ The impact of the ASPs on antibiotic use may differ depending on the prevalence of resistant infections across clinical settings and geographical regions^1^ as well as on available resources.^11^ To date, there is little consolidated evidence on the effectiveness of ASPs in low- and middle-income countries (LMICs), where antimicrobial use is exceptionally high compared with high-income countries (HICs).^11,12^

Moreover, little evidence exists on how targeted interventions can improve the rational use of specific antibiotic classes in different health care contexts. Existing research on ASPs has been mostly restricted to limited comparisons in hospital and intensive care settings.^4,10,13,14,15,16^ It is unclear how ASPs in different contexts affect the consumption of specific antimicrobial agents used in different health care settings. The main objectives of the present review are (1) to provide up-to-date pooled estimates of the association of ASPs with antibiotic consumption and (2) to estimate the differential association of ASPs with the use of different antibiotic classes and across health care and income settings.

## Methods

### Search Strategy

This systematic review and meta-analysis followed the Preferred Reporting Items for Systematic Reviews and Meta-analyses (PRISMA) reporting guideline.^17^ The protocol was registered with PROSPERO (CRD42020206479). We searched PubMed, Web of Science, and Scopus databases from August 1, 2010, to August 1, 2020, for articles on the association of ASPs with antimicrobial consumption (eTable 1 in Supplement 1). Additional studies were identified from the bibliography sections of previous systematic reviews identified in our search. We searched for primary studies conducted with human participants and excluded studies in animals and the environment (eTable 2 in Supplement 1).

### Study Selection

Two independent reviewers (K.Z.Y. and P.T.N.W.) assessed the eligibility of each full-text article; a third reviewer (M.L.) decided cases without consensus. Two of us (K.Z.Y. and P.T.N.W.) reviewed identified articles and the data extraction process as suggested by the PRISMA checklist. P.T.N.W. conducted the quality assessment of all eligible studies. A third author (M.L.) reviewed the articles in doubt, additional references, and data extraction items.

### Risk of Bias Assessment

We used the Effective Public Health Practice Project (EPHPP) quality assessment tool to assess 6 domains of quality: (1) selection bias, (2) design, (3) confounders, (4) blinding, (5) data collection methods, and (6) withdrawal and dropouts.^18^ EPHPP is a widely used assessment tool for quantitative studies designed for systematic literature reviews of effectiveness studies.^19^ The aim of the quality assessment was to evaluate the overall quality of evidence and the risk of bias.^19^ Two independent reviewers rated all articles as strong, moderate, or weak in each domain (eTable 3 in Supplement 1). To avoid potential bias through inappropriate study designs, we only included articles with high study quality, ie, studies that had strong or moderate ratings in at least 5 of 6 domains. Disagreements were discussed by reviewers until consensus was reached. In addition, we assessed publication bias via the Egger test.

### Data Extraction

We extracted the following information from all studies: the aim of the study, country, study design, type of health care facility, study populations, number of health care workers and facilities, pathogens, antibiotic studied, timeline, duration of the interventions, intervention components, and quantitative measure of antibiotic consumption before and after intervention whenever possible. Two separate outcome measures were extracted for preintervention vs postintervention study designs; 4 outcome measures were extracted for randomized trials with clearly defined control and treatment groups. When a study reported both antibiotic-specific consumption measures and an average over all antibiotics, we extracted detailed antibiotic-specific measures. ASPs were defined broadly to include both single-component and multicomponent interventions (eg, a study that implements decision support tools only vs a package combining decision support tools with prospective audit and feedback).

### Statistical Analysis

#### Effect Size Measures

The current literature uses 2 distinct types of outcome measures. First, actual drug consumption is typically measured either as defined daily dose (DDD) per 100 or 1000 patient-days (PDs) or as days of therapy (DOT) per 100 or 1000 PDs. DDD measures drugs administered as multiples of the assumed average maintenance dose per day for a specific patient (typically an adult).^20^ DOT is the number of days of antibiotic therapy administered to a patient, regardless of the number of doses administered or dosage strength.^20^ Since DDD and DOT are conceptually similar, we pooled them in the meta-analysis. We standardized all DDD and DOT measures to 100 PDs. The second outcome often used is the proportion of patients receiving an antibiotic prescription—a separate measure that does not measure drug consumption directly. For each study we calculated 1 of 2 outcomes: (1) the change in antibiotic prescriptions after the intervention compared with before or (2) the rate ratio (RR) of antibiotic consumption after intervention measured in DDD or DOT per 100 PDs compared with the preintervention period. To calculate standard errors of the rate ratios, we calculated log rate ratios as an intermediate step.^21^ Given that these yield asymmetric confidence intervals, we truncated the upper bounds of the intervals at a value of 20.

#### Unit of Analysis and Synthesis Methods

We estimated 3-level meta-analytical models to get pooled average effectiveness estimates.^22^ In contrast to the standard random effects meta-analytical model that accounts for study-level sampling error and between-study heterogeneity, a 3-level model can account for within-study heterogeneity.^22^ With this approach, all reported effect sizes from a single study can be included in the analysis, but multiple effect sizes from the same study contribute less to the overall estimates than single effect sizes from other studies.^23^ The specific weight assigned to each study depends inversely on how strong the correlations are between all effect sizes derived from the same study. Two strongly correlated effect sizes from the same study will both receive lower weights than two weakly correlated effect sizes since they will add little independent information to the pooled effect size. This will then be reflected in a lower study-specific weight. Restricted maximum likelihood models with nested 3-level random-effects were estimated, and Cochran’s *Q* as well as *I*^2^ were computed to assess heterogeneity. R version 4.1.2 (R Project for Statistical Computing) was used to conduct statistical analysis. Statistical significance was set at *P* < .05.

#### Subgroup Analyses

We stratified results based on the following subgroups: HICs and LMICs based on the World Bank income group classification^24^; study settings (primary care practice, pediatric hospital, public hospital); patient settings (outpatient, nursing care, inpatient, intensive care unit [ICU]); antibiotic restriction (restricted or nonrestricted as per individual protocol); and World Health Organization Access, Watch, and Reserve (AWaRe) classification antibiotics 2019.^24,25^ Finally, we stratified results by individual ASP components when it was possible to obtain their individual associations with antibiotic consumption.

## Results

**Figure 1.**
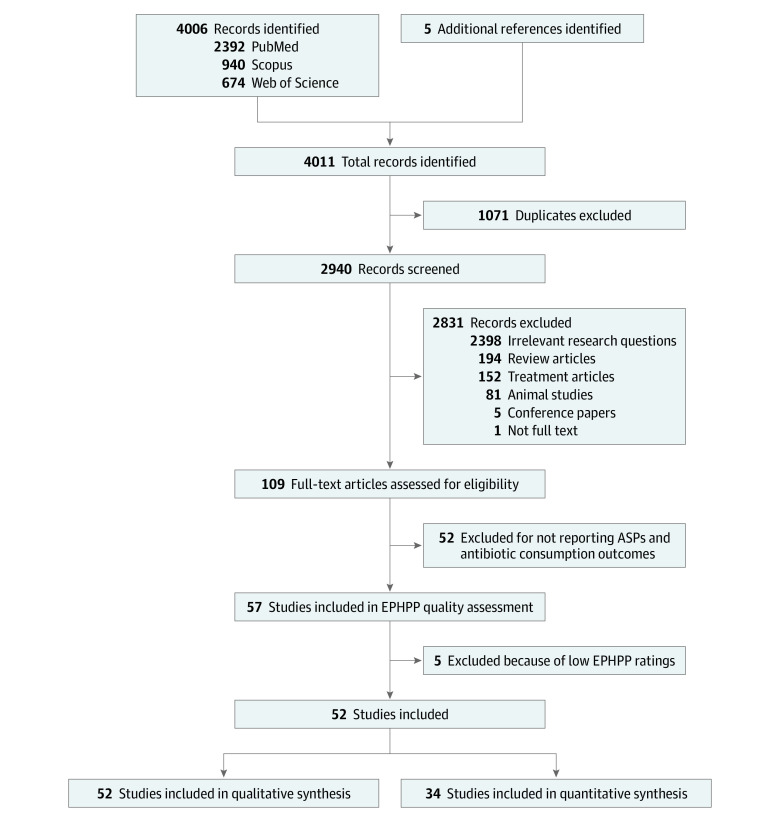
Study Flow Diagram ASP indicates antibiotic stewardship program; EPHPP, Effective Public Health Practice Project*.*

We identified 4011 citations from PubMed, Scopus, and Web of Science; 5 additional articles were obtained from the bibliography of older systematic reviews. After removing duplicates, 2940 unique citations were screened on title and abstract, and 109 citations were included for full-text review. From these, 52 articles^3,5,6,7,9,26,27,28,29,30,31,32,33,34,35,36,37,38,39,40,41,42,43,44,45,46,47,48,49,50,51,52,53,54,55,56,57,58,59,60,61,62,63,64,65,66,67,68,69,70,71,72^ were included in qualitative synthesis, while 34 studies^3,5,7,27,28,29,30,33,36,38,39,40,41,42,43,45,46,47,48,51,52,54,55,57,58,60,61,63,64,65,66,68,69,72^ had sufficient data to be included in the quantitative meta-analysis (Figure 1).

### Characteristics of Included Studies

The final set of studies included in the analysis comprised 19 prospective intervention studies,^6,9,26,27,31,36,37,38,39,42,43,48,60,63,66,70,71^ 12 randomized clinical trials,^7,28,29,34,40,46,50,51,54,55,57,61^ 10 quasi-experimental studies,^5,32,41,44,47,53,62,64,65,68^ 7 nonrandomized controlled trials,^30,33,35,45,49,58,59^ and 4 retrospective cohorts.^3,67,69,72^ Forty studies^3,5,6,7,9,30,31,32,33,34,35,37,38,39,40,42,43,44,45,46,47,49,50,51,52,53,54,55,57,58,59,60,64,65,66,67,68,69,70,72^ were conducted in HICs, and 12 studies^26,27,28,29,36,41,48,56,61,62,63,71^ in LMICs (eTable 4 in Supplement 1). Most studies were conducted in tertiary care hospitals (n = 32)^3,5,9,26,27,31,32,34,36,37,38,39,41,42,43,44,45,52,53,58,59,60,62,63,64,65,67,68,69,70,71,72^ and primary care sites (n = 11).^7,28,29,33,40,47,49,50,54,55,61^ The remaining studies were conducted in general practitioner medical practices (n = 3),^46,51,56^ ICUs (n = 3),^48,66,72^ and nursing homes (n = 3).^30,35,57^ Participants were typically inpatients, including ICU patients (n = 32),^3,5,6,9,26,27,31,32,34,36,37,38,39,40,42,43,46,50,53,58,59,62,63,64,65,66,67,68,69,70,71,72,73,74,75,76^ followed by outpatients (n = 10),^7,28,29,33,47,49,51,54,55,61^ pediatric inpatients (n = 7),^41,44,45,48,52,56,60^ and nursing home residents (n = 3).^30,35,57^ Most studies analyzed ASPs comprising multiple components. It was therefore not possible to estimate the associations between most individual components of ASPs and antibiotic consumption, except for 2 components: (1) training and guidelines and (2) decision support tools (eTable 4 in Supplement 1). The most common components were (1) training and guidelines, ie, training health workers on treatment practices, AMR, and updating guidelines; (2) decision support tools, ie, electronic or paper-based algorithms to assist health workers in treatment decisions; (3) antibiotic restriction, ie, active restrictions on antibiotic use, eg, via preauthorization; (4) prospective audit and feedback, ie, expert physicians or pharmacists review patient cases and the antibiotics they have been prescribed; (5) tracking, ie, monitoring, documenting, and reporting prescription practices and infection and resistance patterns; (6) pharmacy-based interventions, ie, engaging pharmacists to document antibiotic indications, dosage adjustment, and drug interactions and, where needed, to optimize treatment by switching antibiotics; and (7) microbiology-based interventions, ie, antibiotic susceptibility tests to guide decisions (eTable 5 in Supplement 1).

### Pooled Association of ASPs With Antibiotic Consumption

**Figure 2.**
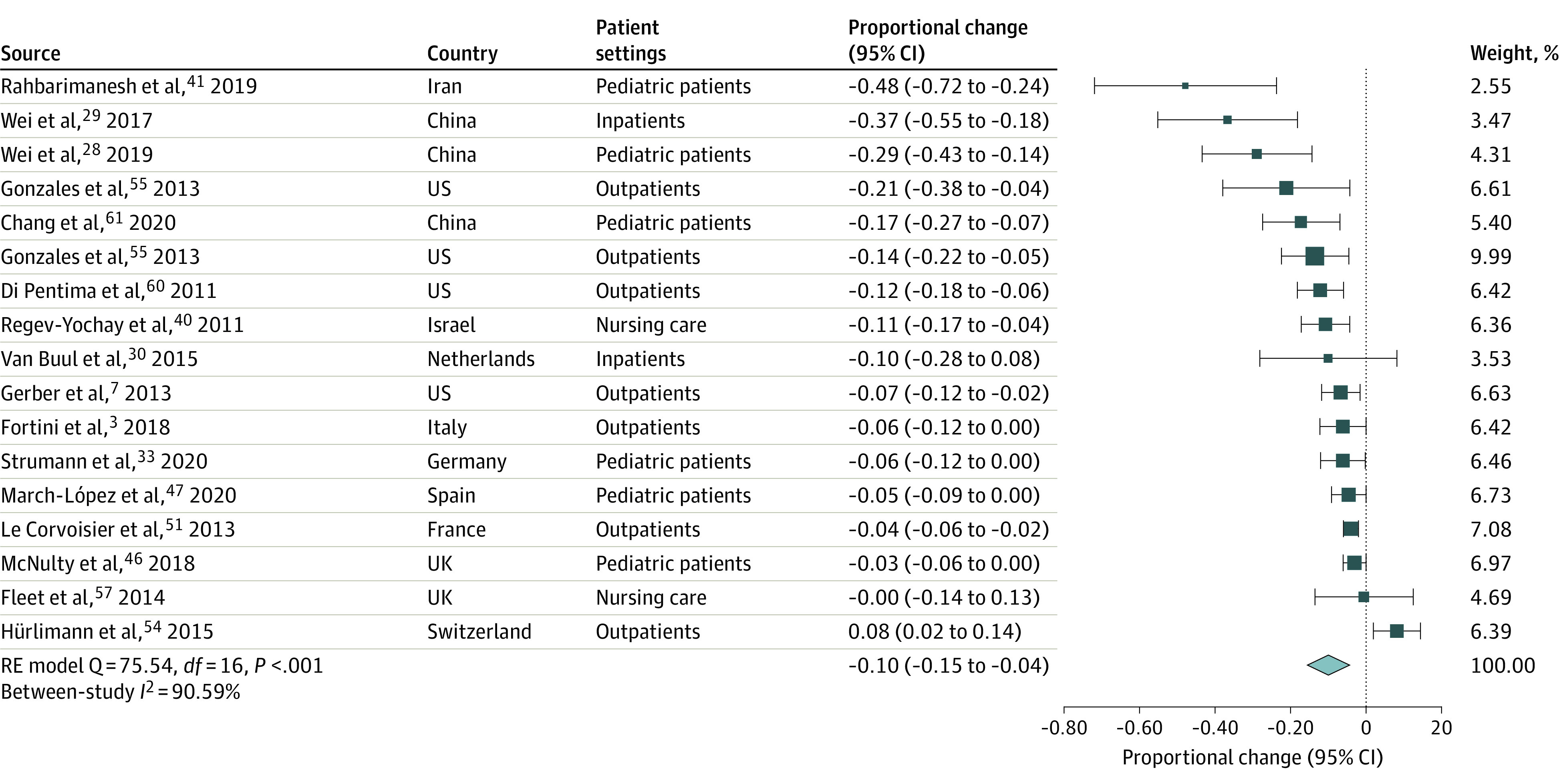
Proportional Change in Antibiotic Prescription, After Compared With Before Intervention Change was calculated as the proportion of all patients who received an antibiotic prescription after the intervention minus the same proportion measured in the preintervention period. For randomized clinical trials, preintervention differences in the proportion of prescriptions between treatment and control groups were subtracted from postintervention differences. A negative effect size indicates that antibiotic stewardship programs were associated with a reduction in antibiotic prescriptions of a magnitude equal to the value of the effect size itself. RE indicates random effects.

**Figure 3.**
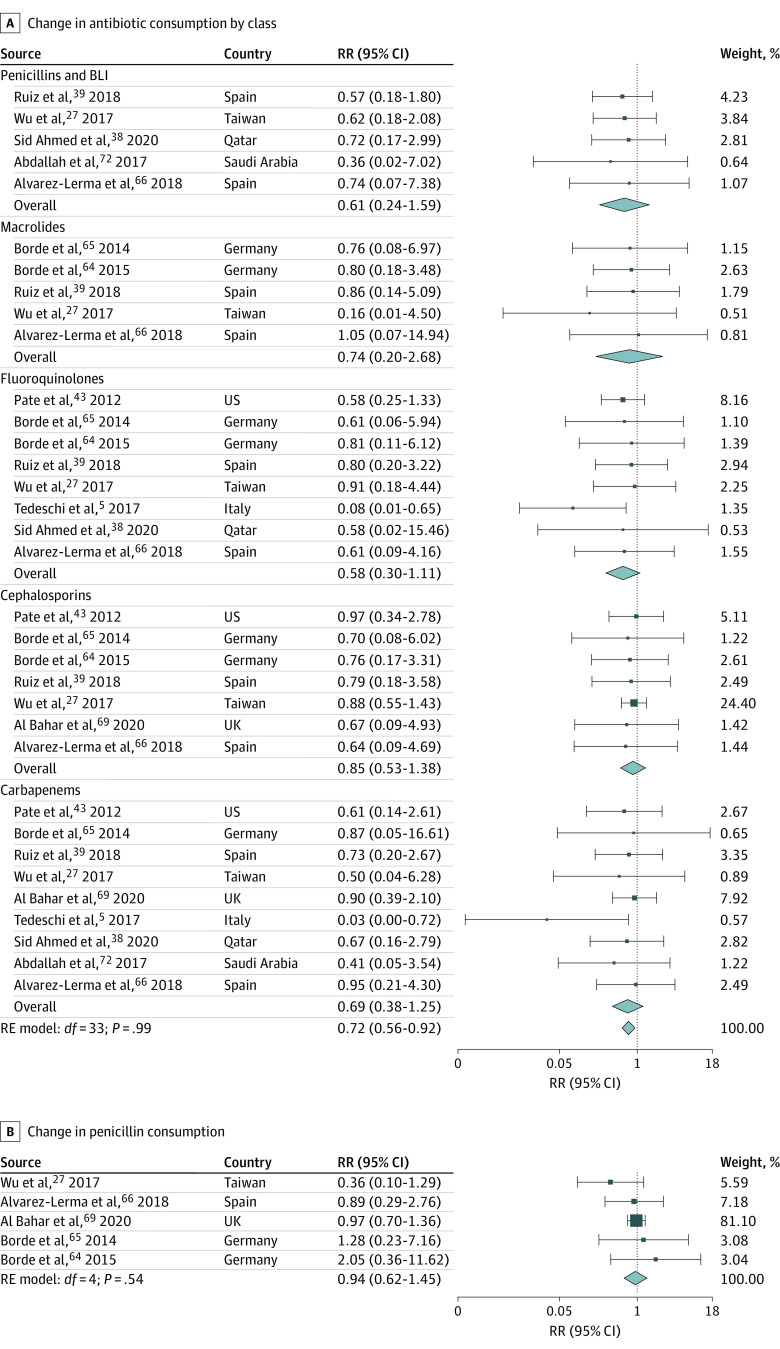
Change in Antibiotic Consumption After vs Before Intervention by Antibiotic Class, in Defined Daily Dose per 100 Patient-Days A, Results stratified by antibiotic classes targeted by antibiotic stewardship programs. B, Penicillins only since their use was either less targeted or even encouraged in some studies. The rate ratio (RR) of antibiotic consumption was obtained by dividing the post-intervention consumption rate measured in defined daily doses per 100 patient-days by the preintervention consumption rate. An RR less than 1 indicates that ASPs were associated with a reduction of 1 − RR% in antibiotic consumption. BLI indicates β-lactamase inhibitor; RE, random-effects.

**Figure 4.**
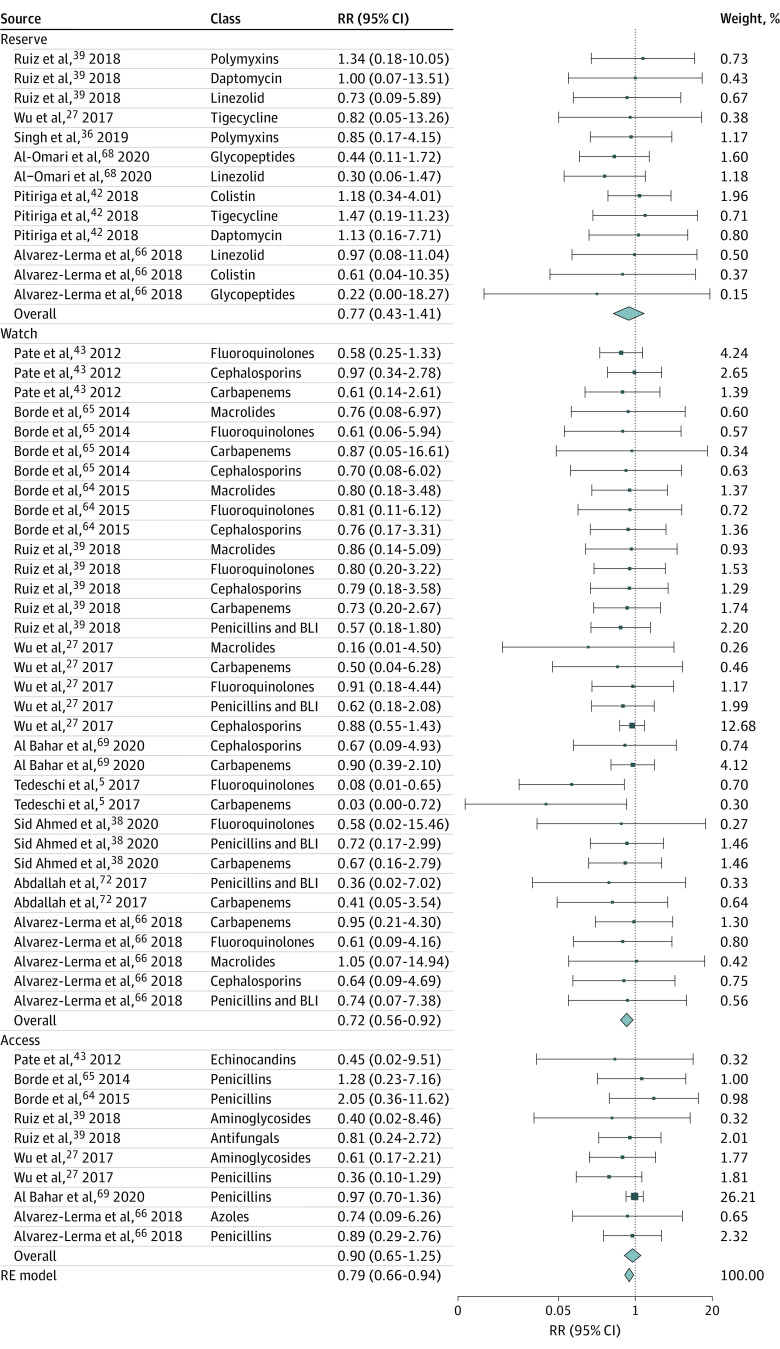
Change in the Consumption of World Health Organization Access, Watch, Reserve Antibiotics After vs Before Intervention, in Defined Daily Dose per 100 Patient-Days Access, Watch, and Reserve categories were obtained from the World Health Organization classification of antibiotics. The rate ratio (RR) of antibiotic consumption was obtained by dividing the postintervention consumption rate measured in defined daily doses per 100 patient-days by the preintervention consumption rate. An RR less than 1 indicates that antibiotic stewardship programs were associated with a reduction of 1 − RR% in antibiotic consumption. RE indicates random effects.

Implementing ASPs was associated with a 10% (95% CI, 4%-15%) decrease in antibiotic prescriptions overall based on 17 estimates (Figure 2),^3,7,28,29,30,33,40,41,46,47,51,54,55,57,60,61^ with substantial heterogeneity across studies (Q = 75.54; *P* < .001). The Egger test suggested possible publication bias (*P* = .007). Five different antibiotic classes from 10 studies reported RRs of antibiotic consumption after intervention measured in DDD per 100 PD compared with the preintervention period. Pooled analysis suggested that, on average, ASPs were associated with a 28% reduction in antibiotic consumption (RR, 0.72; 95% CI, 0.56-0.92; 34 estimates) (Figure 3A).^5,27,38,39,43,64,65,66,69,72^ Evidence for potential publication bias was also found in this subset of studies (Egger test *P* = .001). Stratifying results by broad-spectrum antibiotic classes revealed nonstatistically significant pooled differences between ASPs and consumption. However, these associations were based on a small number of studies, resulting in large confidence intervals, and the direction of the effect sizes was systematic: 33 of 34 RRs suggested a reduction in consumption. All class-specific pooled RRs consistently suggested large reductions in consumption, although the results were not statistically significant: fluoroquinolones (42% reduction), penicillin and β-lactamase inhibitor combinations (39% reduction), carbapenems (31% reduction), macrolides (26% reduction), and cephalosporins (15% reduction) (Figure 3A).^5,27,38,39,43,64,65,66,69,72^ Penicillins were less targeted, with 2 studies actually encouraging their use.^64,65^ No significant change in pooled penicillin consumption was identified following ASP implementation (RR, 0.94; 95% CI, 0.62-1.45; 5 estimates) (Figure 3B).^27,64,65,66,69^ Among studies that reported total antibiotic consumption at a given health facility but did not specify which antibiotic classes were included, results suggested that reductions in consumption followed the implementation of ASPs; however, the pooled effect size was not statistically significant (RR, 0.82; 95% CI, 0.66-1.02; 5 estimates) (eFigure 1 in Supplement 1).^45,48,52,58,63^ Reductions in the use of antibiotics on the WHO’s AWaRe list were also observed, but results were only significant for antibiotics on the Watch list (RR, 0.72; 95% CI, 0.56-0.92; 34 estimates) (Figure 4).^5,27,36,38,39,42,43,64,65,66,68,69,72^

### Heterogeneity of Outcomes Associated With ASPs Across Health Care Settings and Countries

**Figure 5.**
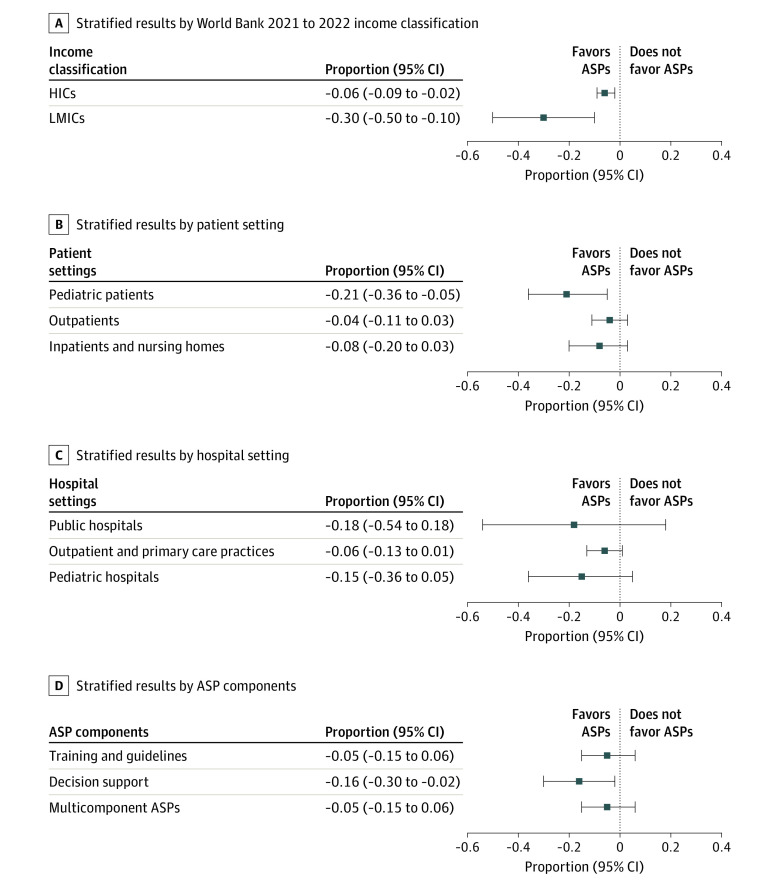
Proportion Change in Antibiotic Prescriptions After vs Before Intervention: Subgroup Analyses Results stratified for the change in the proportion of patients receiving an antibiotic prescription in the postintervention vs preintervention period. The size of each square represents the pooled effect size. A, Stratified results by World Bank 2021 to 2022 income classification. B, Stratified results by patient settings. C, Stratified results by hospital setting. D, Stratified by antimicrobial stewardship program (ASP) components. HIC indicates high-income country; LMIC, low- and middle-income country.

Results stratified by patient setting showed that the largest reductions in antibiotic use were generally found in pediatric care (21% [95% CI, 5% to 36%] reduction; 6 effect sizes) (Figure 5). Antibiotic prescriptions in HICs following ASPs were associated with an average reduction of 6% (95% CI, 2% to 9%; 13 effect sizes) (Figure 5). In contrast, antibiotic consumption in LMICs following ASPs were associated with an average reduction of 30% (95% CI 10% to 50%; 4 effect sizes) (Figure 5). ASPs were not associated with a reduction in antibiotic prescriptions for outpatients (–4%; 95% CI, –11% to 3%; 7 effect sizes)^33,46,47,51,54,55^ or inpatients and nursing home residents (–8%; 95% CI –20% to 3%; 4 effect sizes),^3,30,57,61^ although reductions cannot be ruled out due to the small sample size. Similar nonsignificant results were found across different settings, including public hospitals (–18%; 95% CI, –54% to 18%; 3 effect sizes) and pediatric hospitals (–15%; 95% CI, –36% to 5%; 4 effect sizes) (Figure 5). Decision support tools were associated with a 16% reduction in antibiotic prescriptions (95% CI, 2% to 30%; 3 effect sizes), while no significant association was detected for training and education (–5%; 95% CI, –15% to 6%; 6 effect sizes) and multicomponent ASPs (–5%; 95% CI, –15% to 6%; 8 effect sizes) (Figure 5D). Detailed forest plots for the results in Figure 5 can be found in eFigure 2 in Supplement 1. The stratified results and meta-analysis for antibiotic consumption can also be seen in eFigures 3 and 4 in Supplement 1.

## Discussion

The results of our meta-analysis presented here suggest that ASPs were associated with a 10% reduction in antibiotic prescriptions and a 28% reduction in antibiotic consumption rates. Reductions in consumption were observed across all antibiotic classes, including penicillin and β-lactamase inhibitor combinations, macrolides, fluoroquinolones, cephalosporins, and carbapenems. The only exceptions were penicillins, which is not surprising giving that these are not targeted by all interventions and in some cases even encouraged.^64,65^ ASPs were also associated with reduced consumption of antibiotics on the WHO Watch list, with particularly high risk of selection of bacterial resistance.^73^ In light of concerning increased use of Watch antibiotics globally, this is good news, as it suggests that protecting these drugs through appropriate ASPs is possible.^73^

Subgroup analysis suggests that ASPs were associated with reductions in antibiotic prescriptions in pediatric care, where antibiotic use is particularly high.^74^ Prescriptions for other inpatient, outpatient, and nursing home patients were generally smaller and often not significant.

Moreover, our pooled analysis suggests that ASPs implemented in HICs were associated with reduced antibiotic prescriptions by 6%, echoing findings from previous studies.^10,14^ For the meta-analysis, we only identified 4 studies in LMICs, 3 of which were from China^27,28,29^ and 1 from Iran.^41^ While ASPs were associated with relatively large reductions in prescriptions in LMICs, this must be interpreted with caution due to the small number of studies currently available from LMICs. Uncertainty still remains about the outcomes of ASP in resource-limited settings. One study conducted in a pediatric tertiary hospital in China^56^ suggested that a multicomponent ASP package combining prior authorization, audit and feedback, and pay for performance was more effective than a single strategy. A study in multiple primary care institutions in China^61^ found that physicians’ prescribing behavior did not affect the rate of antibiotic prescriptions, but a computer network-based feedback intervention was associated with significant reductions in antibiotic prescriptions.

A study conducted in 47 small hospitals in South Africa^63^ did not report quantitative estimates of consumption, but it found that introducing pharmacist expertise in a setting with limited infectious disease resources had substantial consequences for antibiotic use and consumption. Overall, the evidence from LMICs remains mixed. Given the challenges involved with the implementation of ASP in LMICs, including often limited availability and access to antibiotics, unavailable diagnostics, and weak adherence to treatment, further research on how to best implement ASPs without compromising the quality of care provided to patients in LMICs is urgently needed.^11,75,76,77,78^ While the present study tried to also analyze the outcome of specific ASP components, the currently available data are not sufficient to assess the relative effectiveness of each component.

### Limitations

This study has limitations. First, the pooled effect sizes cannot be directly interpreted as the causal effect of ASPs on antibiotic prescription or consumption rates since few of the included studies were designed as randomized clinical trials. A control group followed up through the baseline and intervention periods could provide important information on time trends, seasonality, or other factors, including trends in pathogen prevalence or changes in infection control measures that could affect antibiotic consumption. Moreover, as already mentioned, we found very few studies from LMICs. While we may expect the marginal impact of a well-implemented ASP to be larger in an LMIC than in an HIC, the currently available data are not sufficient to assess these differences systematically. Furthermore, our review also did not assess the impacts of stewardship programs on animals and the environment, which are 2 areas that are likely affected and important from one health approach.

## Conclusions

In this systematic review and meta-analysis of the association of ASPs with antimicrobial consumption, ASPs were associated with reduced antibiotic consumption in both hospital and nonhospital settings. Our results show that ASPs can reduce the consumption of WHO Watch group antibiotics with high resistance potential and can potentially contribute to major reductions in antimicrobial consumption in pediatric patients. Overuse and misuse of antibiotics are the main drivers of AMR; reducing antimicrobial consumption through ASPs should thus contribute toward reducing the risk of AMR. This study is limited by the availability of assessments of ASPs in resource-limited settings. Pragmatic randomized clinical trials of ASPs explicitly linking appropriateness of antibiotic utilization to resistant bacterial prevalence as an outcome should therefore be a key research priority. Performance of ASPs might vary considerably in different income settings, and this warrants a particular focus on LMICs where implementation of ASPs could face operational, behavioral, and financial challenges.

## Notes

Supplement 1. **eTable 1.** Search String for the Systematic Review and Meta-analysis **eTable 2.** Inclusion and Exclusion Criteria for Article Screening **eTable 3.** Quality Assessment of the 57 Studies Using the Effective Public Health Practice Project Quality Assessment Tool **eTable 4.** Characteristic of Included Studies in the Systematic Review and Meta-analysis **eTable 5.** Summary of ASP Components Identified in the Included Studies **eFigure 1.** Change in Total Antibiotic Consumption after ASPs (DDD or DOT per 100 Patient-Days) **eFigure 2.** Subgroup Analyses (Antibiotic Prescriptions) **eFigure 3.** Subgroup Analyses (Consumption in DDD per 100 Patient-Days) **eFigure 4.** Meta-analysis Summary (Antibiotic Consumption in DDD per 100 Patient-Days)

Supplement 2.
Data Sharing Statement
