# Supplementary material for: Association Between Antimicrobial Stewardship Programs and Antibiotic Use Globally: A Systematic Review and Meta-Analysis
Source: JAMA Netw Open. 2023 Feb 9;6(2):e2253806. doi: 10.1001/jamanetworkopen.2022.53806 (PMC9912134; doi:10.1001/jamanetworkopen.2022.53806)
Supplement: Supplement 1. — eTable 1. Search String for the Systematic Review and Meta-analysis eTable 2. Inclusion and Exclusion Criteria for Article Screening eTable 3. Quality Assessment of the 57 Studies Using the Effective Public Health Practice Project Quality Assessment Tool eTable 4. Characteristic of Included Studies in the Systematic Review and Meta-analysis eTable 5. Summary of ASP Components Identified in the Included Studies eFigure 1. Change in Total Antibiotic Consumption after ASPs (DDD or DOT per 100 Patient-Days) eFigure 2. Subgroup Analyses (Antibiotic Prescriptions) eFigure 3. Subgroup Analyses (Consumption in DDD per 100 Patient-Days) eFigure 4. Meta-analysis Summary (Antibiotic Consumption in DDD per 100 Patient-Days) [file jamanetwopen-e2253806-s001.pdf]

Supplemental Online Content

Zay Ya K, Win PTN, Bielicki J, Lambiris M, Fink G. Association between antimicrobial stewardship programs and antibiotic use globally: a systematic review and meta-analysis. *JAMA Netw Open.* 2023;6(2):e2253806. doi:10.1001/jamanetworkopen.2022.53806

eTable 1. Search String for the Systematic Review and Meta-analysis

eTable 2. Inclusion and Exclusion Criteria for Article Screening

eTable 3. Quality Assessment of the 57 Studies Using the Effective Public Health Practice Project Quality Assessment Tool

eTable 4. Characteristic of Included Studies in the Systematic Review and Meta-analysis

eTable 5. Summary of ASP Components Identified in the Included Studies

eFigure 1. Change in Total Antibiotic Consumption after ASPs (DDD or DOT per 100 Patient-Days)

eFigure 2. Subgroup Analyses (Antibiotic Prescriptions)

eFigure 3. Subgroup Analyses (Consumption in DDD per 100 Patient-Days)

eFigure 4. Meta-analysis Summary (Antibiotic Consumption in DDD per 100 Patient-Days)

This supplemental material has been provided by the authors to give readers additional information about their work.

© 2023 Zay Ya K et al. *JAMA Network Open.*

eTable 1. Search String for the Systematic Review and Meta-analysis

Keywords in combination
"drug resistance, microbial"[MeSH Terms] OR Antibiotic resistance OR Antimicrobial Resistance OR Microbial Drug Resistance OR Antimicrobial Drug Resistance OR Multiple Drug Resistance OR Antibiotic Use OR Antibiotic Prescription OR Antibiotic Consumption<br>AND<br>Polic* OR Intervention*<br>AND<br>Impact* OR Evaluate* OR Assess* OR Effect*

© 2023 Zay Ya K et al. *JAMA Network Open.*

eTable 2. Inclusion and Exclusion Criteria for Article Screening

Inclusion criteria<br>➢ Studies in humans<br>➢ Reports on policy and interventions designed to reduce antibiotic use or antimicrobial resistance<br>➢ Primary studies<br>➢ Full text availability<br>➢ Report in English<br>➢ Studies within 1 Aug 2010 to 1 Aug 2020
Exclusion criteria<br>➢ Studies in animals<br>➢ Reports not relating with AMR policies and interventions<br>➢ Review, expert opinions, conference abstracts posters and newspaper articles<br>➢ Report in other language<br>➢ Study before 1 Aug 2010

© 2023 Zay Ya K et al. *JAMA Network Open.*

eTable 3. Quality Assessment of the 57 Studies Using the Effective Public Health Practice Project Quality Assessment Tool

Author	Study type	Selection bias	Study design	Confounders	Blinding	Data collection methods	Withdrawal and drop-outs	Global rating
Sloane et al	Controlled trial	**S**	**S**	**S**	**M**	**S**	**S**	**M**
Alvarez-Lerma et al	Prospective interventional cohort	**S**	**M**	**W**	**M**	**S**	**M**	**M**
Gonzales et tal	RCT	**S**	**S**	**S**	**M**	**S**	**S**	**S**
Cross et tal	Cluster-randomized	**M**	**M**	**W**	**M**	**S**	**M**	**M**
Tedeschi et al	Quasi-experimental	**M**	**M**	**W**	**M**	**S**	**M**	**M**
Aldeyab, M. A.	Retrospective interventional cohort	**M**	**M**	**S**	**M**	**S**	**M**	**M**
Brink et al	Prospective interventional cohort	**S**	**M**	**S**	**M**	**S**	**M**	**S**
Fortini et al	Retrospective interventional cohort	**S**	**M**	**S**	**M**	**S**	**M**	**S**
Rahbarimanesh et al	Quasi-experimental	**S**	**M**	**M**	**W**	**S**	**S**	**M**
Pitiriga et al	Prospective interventional cohort	**M**	**M**	**W**	**M**	**S**	**M**	**M**
Ruiz et al	Prospective interventional cohort	**S**	**M**	**S**	**W**	**S**	**M**	**M**
Elligsen et al	Controlled trial	**S**	**S**	**S**	**M**	**S**	**S**	**S**
Dik, J. W. H et al	Controlled trial	**S**	**S**	**S**	**M**	**S**	**S**	**S**
Sikkens et al	Prospective interventional cohort	**S**	**M**	**M**	**W**	**S**	**M**	**M**
Di Pentima et al	Prospective interventional cohort	**S**	**M**	**M**	**W**	**S**	**M**	**M**
Strumann et al	Controlled trial	**M**	**S**	**S**	**W**	**S**	**M**	**M**
Wu, C. T et al	Cohort	**S**	**M**	**S**	**W**	**S**	**S**	**M**
Chang et al	RCT	**S**	**S**	**S**	**M**	**S**	**S**	**S**
Wei, X et al	RCT	**S**	**S**	**S**	**M**	**S**	**S**	**S**
Gerber, J. S et al	RCT	**S**	**S**	**S**	**M**	**S**	**S**	**S**
Gong et al	Prospective interventional cohort	**S**	**M**	**S**	**M**	**S**	**M**	**S**

© 2023 Zay Ya K et al. *JAMA Network Open.*

W. van Buul et al	Controlled trial	**S**	**S**	**S**	**M**	**S**	**M**	**S**
Llor, C et al	Controlled trial	**S**	**S**	**S**	**M**	**S**	**S**	**S**
Llor C et a;l	Cohort	**S**	**M**	**S**	**W**	**W**	**M**	**W**
Little et al	RCT	**S**	**S**	**S**	**S**	**S**	**S**	**S**
McNulty et al	RCT	**S**	**S**	**S**	**S**	**S**	**S**	**S**
March-López et al	Quasi-experimental	**S**	**M**	**S**	**W**	**S**	**M**	**M**
Newland et al	Controlled trial	**M**	**S**	**S**	**M**	**M**	**M**	**S**
Craft et al	Cohort	**M**	**M**	**M**	**W**	**M**	**W**	**W**
Khdour et al	Prospective interventional cohort	**M**	**M**	**W**	**M**	**M**	**M**	**M**
Talpaert et al	Quasi-experimental	**M**	**M**	**W**	**M**	**S**	**M**	**M**
Fleet et al	RCT	**S**	**S**	**S**	**S**	**S**	**M**	**S**
Stenehjem et al	RCT	**S**	**S**	**M**	**M**	**S**	**M**	**S**
Ouldali et al	Quasi-experimental	**M**	**M**	**M**	**M**	**S**	**M**	**S**
Zhou, Y et al	Prospective interventional cohort	**M**	**M**	**M**	**M**	**S**	**W**	**M**
Abubakar et al	Prospective interventional cohort	**S**	**M**	**M**	**W**	**S**	**M**	**M**
Magedanz et al	Cohort	**M**	**M**	**W**	**W**	**M**	**M**	**W**
Singh et al	Prospective interventional cohort	**S**	**M**	**M**	**W**	**M**	**M**	**M**
Tavares et al	Prospective interventional cohort	**S**	**M**	**M**	**M**	**M**	**M**	**S**
Borde et al	Quasi-experimental	**S**	**M**	**M**	**W**	**M**	**M**	**M**
Nitsch-Osuch et a;	Cohort	**S**	**M**	**W**	**W**	**M**	**M**	**W**
Lu, C et al	Prospective interventional cohort	**M**	**M**	**M**	**W**	**M**	**M**	**M**
Hürlimann et al	RCT	**S**	**S**	**S**	**M**	**S**	**M**	**S**
Le Corvoisier et al	RCT	**M**	**S**	**S**	**S**	**S**	**S**	**S**
Jenkins et al	Quasi-experimental	**S**	**M**	**M**	**M**	**S**	**W**	**M**
Wei, X. et al	Cluster randomized	**S**	**S**	**S**	**M**	**S**	**S**	**S**
Kreitmeyr et al	Prospective interventional cohort	**S**	**M**	**S**	**M**	**S**	**S**	**S**

© 2023 Zay Ya K et al. *JAMA Network Open.*

Butt et al	Quasi-experimental	**S**	**M**	**S**	**W**	**S**	**S**	**S**
Regev-Yochay et al	Cluster randomized	**S**	**S**	**S**	**M**	**S**	**S**	**S**
Adhikari et al	Prospective interventional cohort	**S**	**M**	**S**	**M**	**S**	**M**	**S**
Onorato et al	Prospective interventional cohort	**S**	**M**	**W**	**M**	**S**	**M**	**M**
Al Bahar et al	Retrospective interventional cohort	**S**	**M**	**M**	**M**	**S**	**M**	**S**
Borde et al	Quasi-experimental	**S**	**S**	**S**	**S**	**S**	**S**	**S**
Pate et al	Prospective interventional cohort	**S**	**S**	**S**	**S**	**S**	**S**	**S**
Abdallah et al	Retrospective interventional cohort	**S**	**S**	**S**	**S**	**S**	**S**	**S**
Sid Ahmed et al	Prospective interventional cohort	**S**	**S**	**S**	**S**	**S**	**S**	**S**
Al-Omari et al	Quasi-experimental	**S**	**S**	**S**	**S**	**S**	**S**	**S**

In each component, a rating of strong, moderate, weak has to be assigned according to rating guidelines and dictionaries. For global rating of each paper, “Strong” rating was given when there are no weak ratings in all components, “Moderate” for one weak rating and “Weak” for two or weak ratings in one of assessment. Only articles with “Strong” and “Moderate” ratings were included in our analysis. We only included articles with high study quality that had strong or moderate ratings in at least 5 out of the 6 domains.

© 2023 Zay Ya K et al. *JAMA Network Open.*

eTable 4. Characteristic of Included Studies in the Systematic Review and Meta-analysis

Authors	Year of publication	Study design	Country	Study settings	Study population	Interventions	Reduction in antibiotic consumption
Sloane et al	2020	Controlled trial	USA	community Nursing Homes	nursing homes, nursing staff and medical care providers in nursing homes	antimicrobial stewardship programmes	Yes
Alvarez-Lerma et al	2018	Prospective interventional cohort	Spain	ICU in Acute-care teaching hospital	patients in the ICUs of a 400-bed acute-care teaching hospital	antimicrobial stewardship programmes	Yes
Gonzales et al	2013	RCT	USA	primary care practices	uncomplicated acute bronchitis patients in primary care	electronic decision support	Yes
Tedeschi et al	2017	Quasi-experimental	Italy	rehabilitation hospital	patients with spinal-cord injuries	antimicrobial stewardship program	Yes
Aldeyab, M. A.	2012	Retrospective interventional cohort	Northern Ireland	one hospital within the Trust	patients with *Clostridium difficile* infection	hospital antibiotic policy	Yes
Brink et al	2016	Prospective interventional cohort	South Africa	private hospitals	patients in private hospital	antimicrobial stewardship programmes	Yes
Fortini et al	2018	Retrospective interventional cohort	Italy	mid-sized acute care hospital	patients Internal Medicine ward	antimicrobial stewardship programmes	Yes
Rahbarimanesh et al	2019	Quasi-experimental	Iran	children's hospital	paediatric patients	antimicrobial stewardship programmes	Yes
Pitiriga et al	2018	Prospective interventional cohort	Greece	modern medicine hospital	Patients from cardiac surgery, intensive care unit (ICU), orthopaedic surgery, oncology, neurosurgery, urology and acute medical/surgical care	antimicrobial stewardship programmes	No
Ruiz et al	2018	Prospective interventional cohort	Spain	medical ICU in a tertiary hospital	patients in ICU	antimicrobial stewardship programmes	Yes
Elligsen et al	2012	Controlled trial	Canada	single tertiary care centre with 3 intensive care units	medical and surgical patients as well as regional trauma patients; cardiac and vascular surgery patients; and burn patients	antimicrobial stewardship programmes	Yes
Dik, J. W. H et al	2015	Controlled trial	Netherland	university medical centre urology ward	patients in urology ward	antimicrobial stewardship programmes	Yes

© 2023 Zay Ya K et al. *JAMA Network Open.*

Sikkens et al	2017	Prospective interventional cohort	Netherland	tertiary care medical centre and general teaching hospital	patients in 7 clinical departments	training and guidelines	Yes
Di Pentima et al	2011	Prospective interventional cohort	USA	tertiary care academic paediatric hospital	paediatric oncology patients and patients who were receiving stem cell transplantations	antimicrobial stewardship programmes	Yes
Strumann et al	2020	Controlled trial	Germany	primary care physicians in private practices	URTI cases	training and guidelines	Yes
Wu, C. T et al	2017	Cohort	Taiwan	general hospital	all patients admitted to Nan Men General Hospital	antimicrobial stewardship programmes	Yes
Chang et al	2020	RCT	China	primary care institutions	patients in community health service centres	computerized decision support	Yes
Wei, X et al	2017	RCT	China	primary care hospitals	paediatric outpatients	antimicrobial stewardship programmes	Yes
Gerber, J. S et al	2013	RCT	USA	paediatric primary care sites	paediatric outpatients	antimicrobial stewardship programmes	Yes
Gong et al	2016	Prospective interventional cohort	China	tertiary paediatric hospital	paediatric outpatients	antimicrobial stewardship programmes	Yes
W. van Buul et al	2015	Controlled trial	Netherland	nursing homes and residential care facilities	nursing homes	antimicrobial stewardship programmes	No changes in antibiotic use
Llor, C et al	2011	Controlled trial	Spain	primary care centres	all cases of pharyngitis	antimicrobial stewardship programmes	Yes
Little et al	2013	RCT	Six European countries	primary-care practices	patients with LRTIs	internet-based training	Yes
McNulty et al	2018	RCT	United Kingdom	GP medical practices	patients in general practices	training and guidelines	Yes
March-López et al	2020	Quasi-experimental	Spain	primary health care	PHC patients	antimicrobial stewardship programmes	Yes
Newland et al	2012	Controlled trial	USA	tertiary care children's hospital	paediatric patients	antimicrobial stewardship programmes	Yes
Khdour et al	2018	Prospective interventional cohort	Northern Ireland	tertiary hospital including	ICU and any antimicrobial drug administered patients	antimicrobial stewardship programmes	Yes
Talpaert et al	2011	Quasi-experimental	United Kingdom	acute general hospital	patients in an acute hospital	antimicrobial stewardship programmes	Yes

© 2023 Zay Ya K et al. *JAMA Network Open.*

Fleet et al	2014	RCT	United Kingdom	nursing homes	nursing homes	antimicrobial stewardship programmes	Yes
Stenehjem et al	2018	RCT	USA	critical access hospitals with paediatric units and ICU	patients in 15 small hospitals	antimicrobial stewardship programmes	No
Ouldali et al	2017	Quasi-experimental	France	paediatric patients with ARTI diagnosis	ARTI	treatment guidelines	Yes
Zhou, Y et al	2015	Prospective interventional cohort	China	department of Urology	patients with clean operation	antimicrobial stewardship programmes	Yes
Abubakar et al	2019	Prospective interventional cohort	Nigeria	obstetrics and gynaecology settings	women who had elective and emergency obstetric and gynaecologic surgeries	antimicrobial stewardship programmes	Yes
Singh et al	2019	Prospective interventional cohort	India	academic large hospital	patients in surgical, medical units and critical care	antimicrobial stewardship programmes	Yes
Tavares et al	2018	Prospective interventional cohort	Portugal	university hospital	patients in tertiary care public teaching hospital	antimicrobial stewardship programmes	Yes
Borde et al	2015	Quasi-experimental	Germany	university hospital centre	non-trauma emergency patients	antimicrobial stewardship programmes	Yes
Lu, C et al	2019	Prospective interventional cohort	China	neonatal ICU	infants who received antibiotics during their hospital stay	antimicrobial stewardship programmes	Yes
Hürlimann et al	2015	RCT	Switzerland	primary care physicians	upper RTIs, lower RTIs	treatment guidelines	Yes
Le Corvoisier et al	2013	RCT	France	GPs with a practice in three counties	patients treated by GPs	interactive workshop and educational seminar	Yes
Jenkins et al	2015	Quasi-experimental	USA	public safety net hospital	inpatients	antimicrobial stewardship programmes	Yes
Wei, X. et al	2019	Cluster randomized	China	primary care facilities in rural counties	children with URTIs	interactive workshop and educational seminar	Yes
Kreitmeyr et al	2017	Prospective interventional cohort	Germany	academic children's hospital	paediatric patients	antimicrobial stewardship programmes	Yes
Butt et al	2019	Quasi-experimental	Pakistan	tertiary care hospital	patients with clean/clean contaminated surgeries from three different surgery wards, general,	pharmacist's educational intervention	Yes

© 2023 Zay Ya K et al. *JAMA Network Open.*

					orthopaedic and gynaecology		
Regev-Yochay et al	2011	Cluster randomized	Israel	primary care paediatric solo practices	children in a community setting	antimicrobial stewardship programmes	Yes
Adhikari et al	2018	Prospective interventional cohort	Australia	medical-surgical tertiary Australian adult ICU	patients in tertiary referral hospital level-6 ICU	antimicrobial stewardship programmes	Yes
Onorato et al	2020	Prospective interventional cohort	Italy	ICUs of an acute-care teaching hospital.	patients in ICU	antimicrobial stewardship programmes	Yes
Al Bahar et al	2020	Retrospective interventional cohort	United Kingdom	teaching hospital	patients in a tertiary care hospital	computerised decision support	Yes
Borde et al	2014	Quasi-experimental	Germany	academic teaching hospital and tertiary care referral centre	patients in a tertiary care hospital	antimicrobial stewardship programmes	Yes
Pate	2012	Prospective interventional cohort	USA	long-term acute care hospital	patients in long-term acute care hospital	antimicrobial stewardship programmes	Yes
Abdallah	2017	Retrospective interventional cohort	Saudi Arabia	tertiary care centre particularly spinal and neurosurgery	patients in adult ICU	antimicrobial stewardship programmes	Yes
Sid Ahmed	2020	Prospective interventional cohort	Qatar	acute care hospital	patient in an acute care hospital	antimicrobial stewardship programmes	Yes
Al-Omari	2020	Quasi-experimental	Saudi Arabia	tertiary private hospitals	adult inpatients	antimicrobial stewardship programmes	Yes

© 2023 Zay Ya K et al. *JAMA Network Open.*

eTable 5. Summary of ASP Components Identified in the Included Studies

ASP component	component description
Training and Guidelines	• Training on antibiotic use in formal and informal settings, messaging through posters, flyers, newsletters, or electronic communication to health care providers/service providers, e.g. training on antibiotic use, internet-based training<br>• Educating prescribers, pharmacists, and nurses about adverse reactions from antibiotics, antibiotic resistance, and optimal prescribing e.g. interactive workshop and educational seminar<br>• Developing or updating guideline and protocol about appropriate antibiotic use e.g. develop community-acquired pneumonia guidelines for hospitalists.
Decision support tools	• Decision support through electronic or paper-based strategies for antibiotic use e.g. electronic-based treatment algorithm or a poster with a clinical algorithm
Antibiotic restriction	• Restricting antibiotic use by interventions, such as preauthorization, requires prescribers to gain approval before using certain antibiotics. e.g. preauthorization through an electronic order entry system or ID physician.
Prospective audit and feedback	• An external review of antibiotic therapy by antibiotic experts (usually physicians and/or pharmacists), e.g. case-by-case review of patients prescribed antibiotics by an infectious diseases (ID) physician.
Tracking	• Monitoring and evaluation of antibiotic prescribing and other vital outcomes (antibiotic prescribing and outcome tracking systems) and reporting prescription practices, infection and resistance patterns, e.g. monitoring *C. difficile* infection and resistance patterns.
Pharmacy-based Interventions	• The engagement of pharmacists in ASPs to improve antibiotic use. The pharmacist's role in ASPs is to document antibiotic indications, dosage adjustment, and duplicative antibiotic therapy alerts and to monitor antibiotic-related drug interactions and adverse effects e.g. clinical pharmacist provides a notification to switch antibiotic therapy.
Microbiology-based interventions	• Antimicrobial susceptibility testing results to show antibiotics that are in line with hospital/clinical treatment guidelines or ASPs and to help providers in clinical decision making with microbiology report e.g. antibiotic culture and sensitivity test report.

© 2023 Zay Ya K et al. *JAMA Network Open.*

eFigure 1. Change in Total Antibiotic Consumption after ASPs (DDD or DOT per 100 Patient-Days)

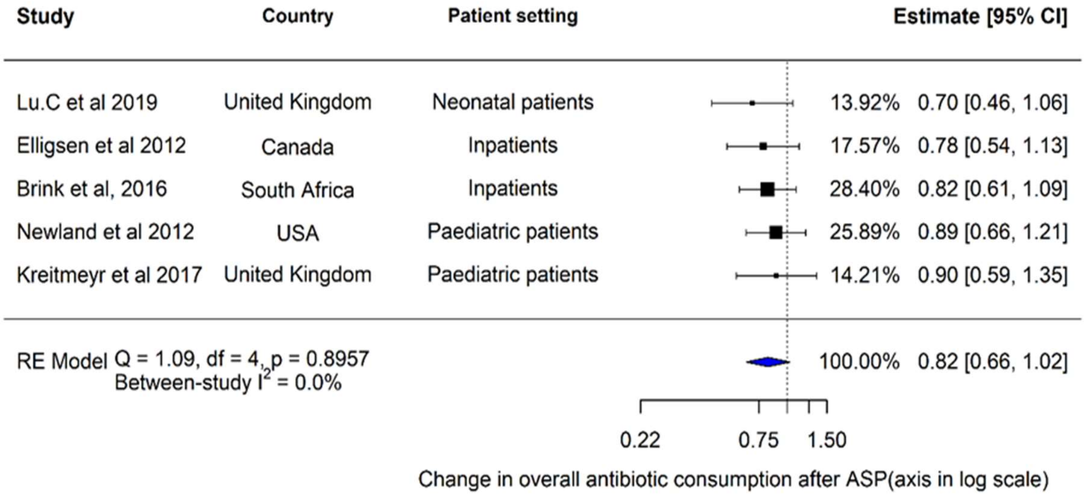

Figure 1 shows the average change in antibiotic consumption post- compared to pre-intervention. RR:rate ratio. The rate ratio (RR) of antibiotic consumption was obtained by dividing the post-intervention consumption rate measured in DDD or DOT per 100 PD by the pre-intervention consumption rate. A rate ratio below the value of 1 indicates that ASPs are associated with a reduction of (1-RR)% in antibiotic consumption. Numbers quoted in percentage terms are the weights assigned to each effect size by the three-level random effects model. 95% confidence intervals are included in brackets. No significant reduction in consumption was measured among studies that reported consumption pooled across antibiotics (RR=0.82, 95% CI [0.66 to 1.02]; 5 estimates).

© 2023 Zay Ya K et al. *JAMA Network Open.*

eFigure 2. Subgroup Analyses (Antibiotic Prescriptions)

A Forest plot of included studies stratified by patient settings (Antibiotic prescriptions)

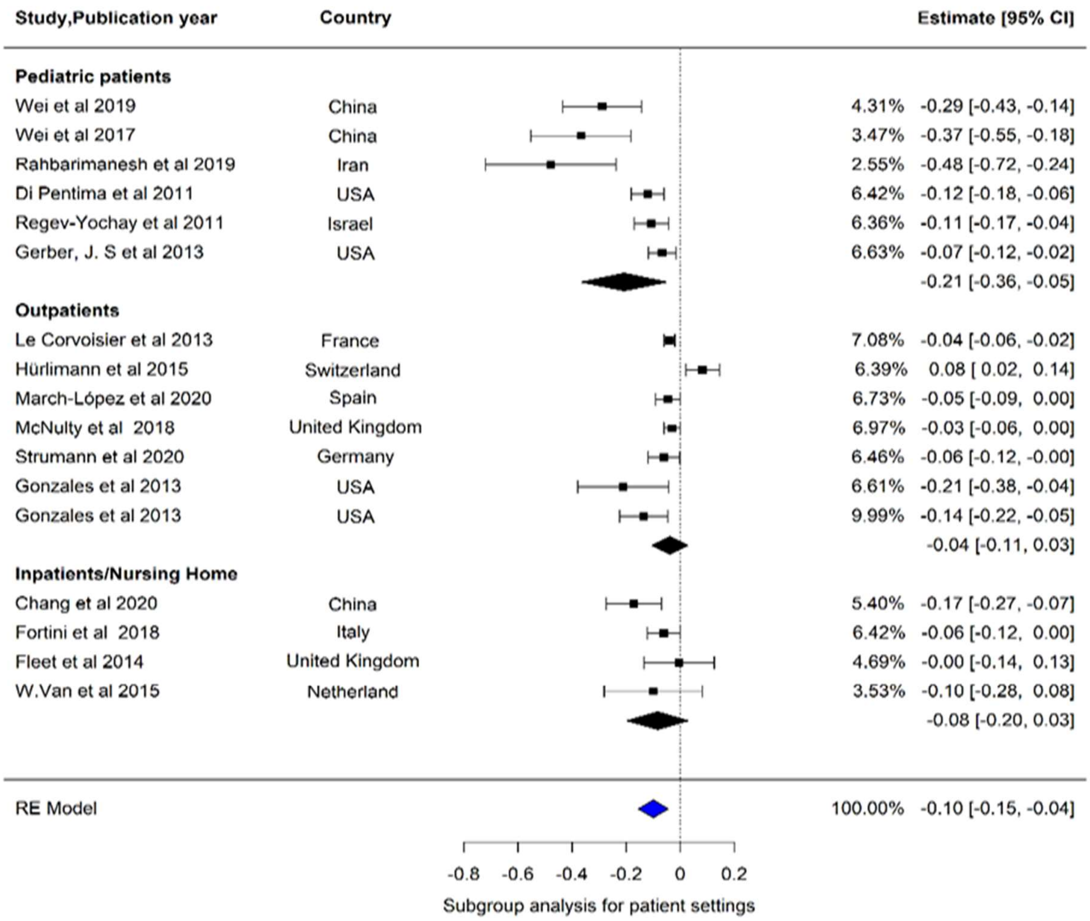

Figure 2a shows the stratified results for the average change in the proportion of patients receiving an antibiotic prescription in the post-intervention compared to the pre-intervention period. This was calculated as the proportion of all patients that received an antibiotic prescription post-intervention minus the same proportion measured in the pre-intervention period. For randomised controlled trials, pre-intervention differences in the proportion of prescriptions between treatment and control groups were subtracted from post-intervention differences. A negative effect size indicates that ASPs are associated with a reduction in antibiotic prescriptions of magnitude equal to the value of the effect size itself. Numbers quoted in percentage terms are the weights assigned to each effect size by the three-level random effects model. 95% confidence intervals are included in brackets.

© 2023 Zay Ya K et al. *JAMA Network Open.*

B: Forest plot of included studies stratified by income classification (Antibiotic prescriptions)

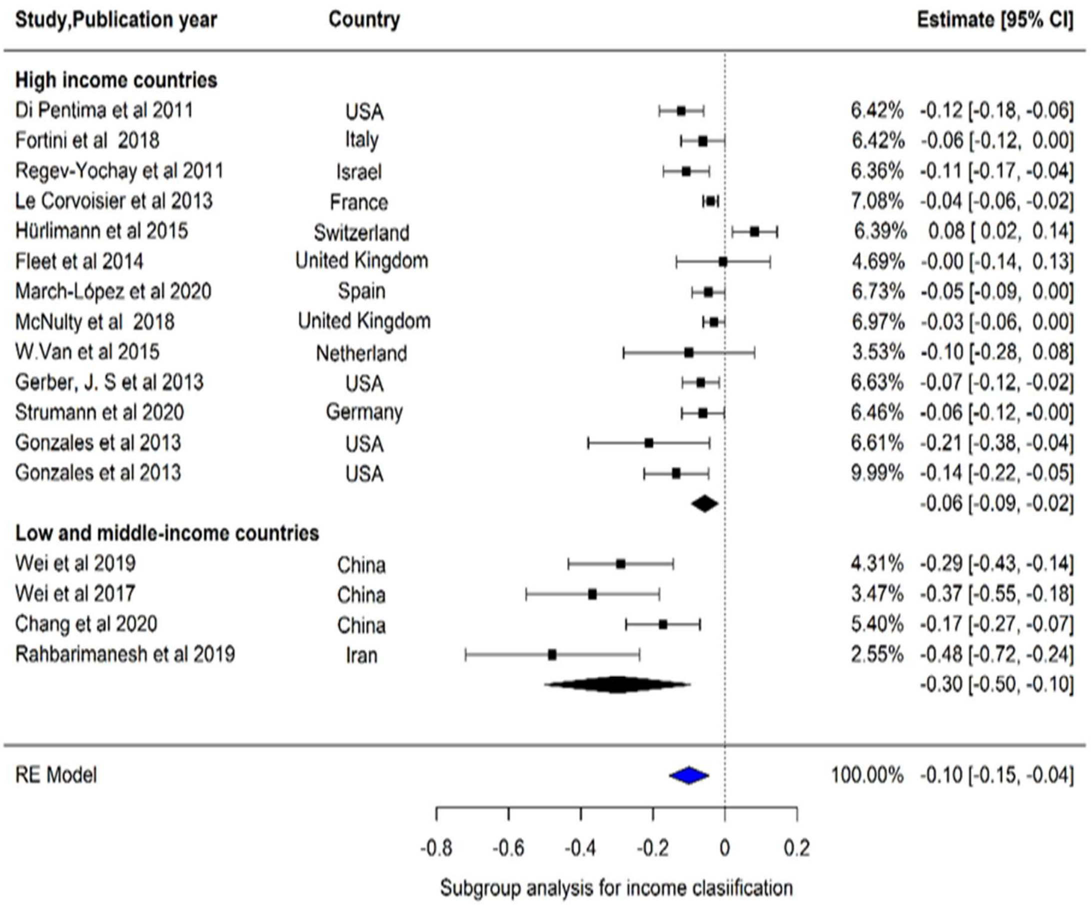

Figure 2b shows the stratified results for the average change in the proportion of patients receiving an antibiotic prescription in the post-intervention compared to the pre-intervention period. This was calculated as the proportion of all patients that received an antibiotic prescription post-intervention minus the same proportion measured in the pre-intervention period. For randomised controlled trials, pre-intervention differences in the proportion of prescriptions between treatment and control groups were subtracted from post-intervention differences. A negative effect size indicates that ASPs are associated with a reduction in antibiotic prescriptions of magnitude equal to the value of the effect size itself. Numbers quoted in percentage terms are the weights assigned to each effect size by the three-level random effects model. 95% confidence intervals are included in brackets.

© 2023 Zay Ya K et al. *JAMA Network Open.*

C: Forest plot of included studies stratified by study settings (Antibiotic prescriptions)

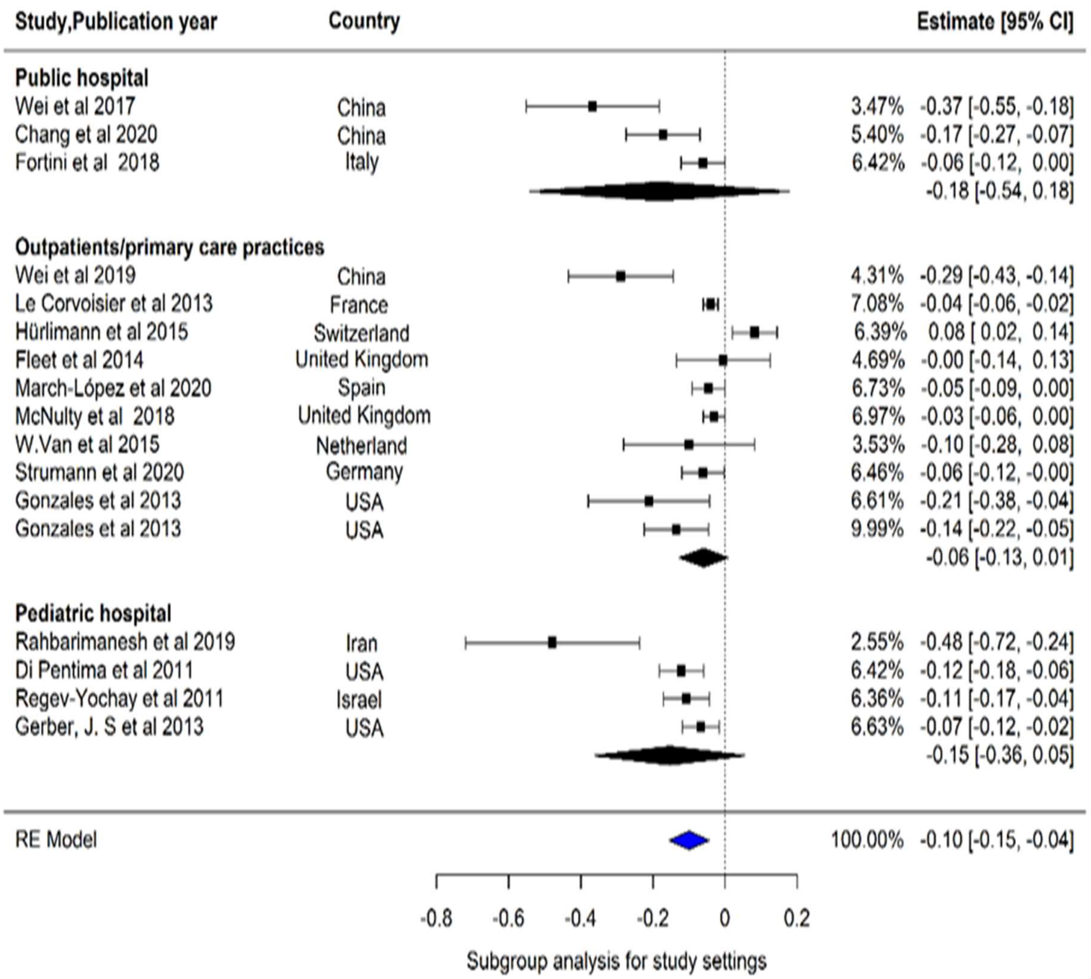

Figure 2c shows the stratified results for the average change in the proportion of patients receiving an antibiotic prescription in the post-intervention compared to the pre-intervention period. This was calculated as the proportion of all patients that received an antibiotic prescription post-intervention minus the same proportion measured in the pre-intervention period. For randomised controlled trials, pre-intervention differences in the proportion of prescriptions between treatment and control groups were subtracted from post-intervention differences. A negative effect size indicates that ASPs are associated with a reduction in antibiotic prescriptions of magnitude equal to the value of the effect size itself. Numbers quoted in percentage terms are the weights assigned to each effect size by the three-level random effects model. 95% confidence intervals are included in brackets.

D: Forest plot of included studies stratified by intervention types (Antibiotic prescriptions)

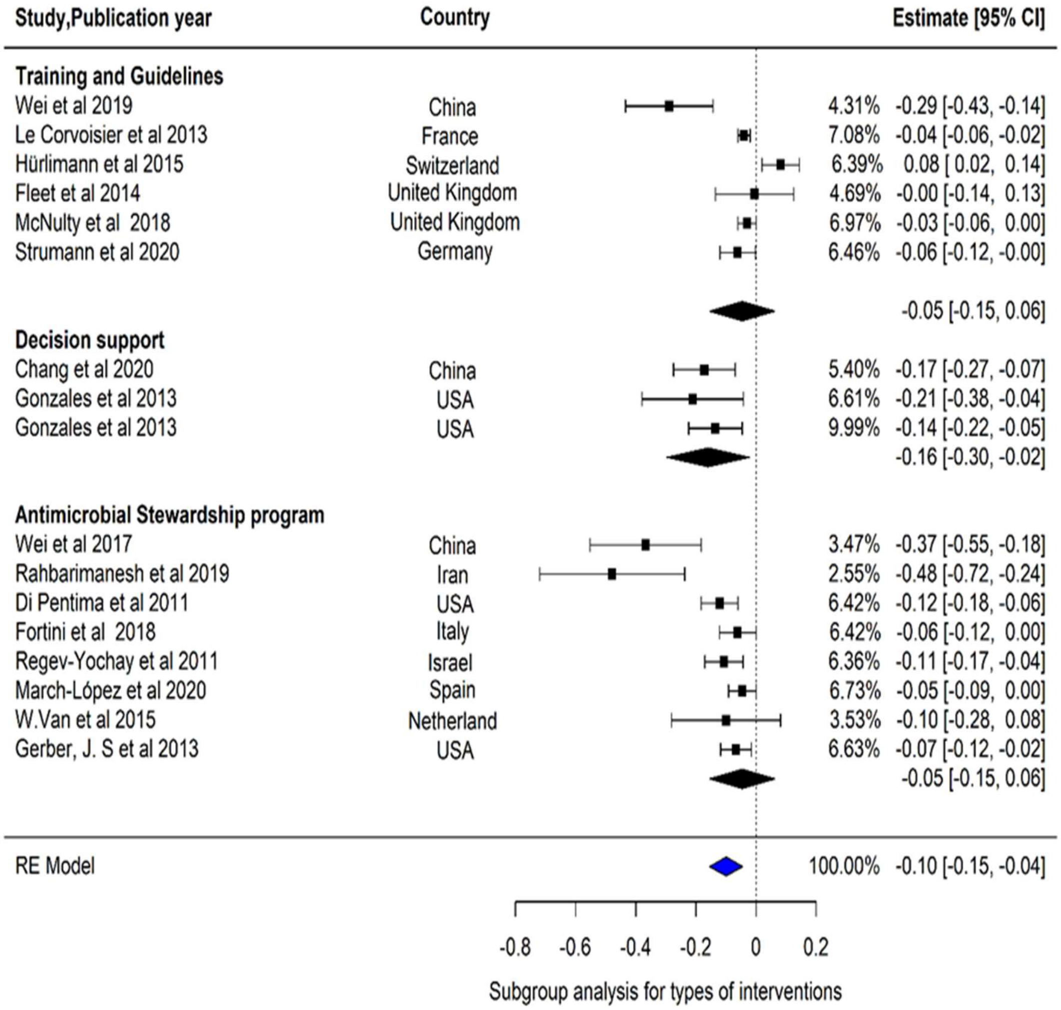

Figure 2d shows the stratified results for the average change in the proportion of patients receiving an antibiotic prescription in the post-intervention compared to the pre-intervention period. This was calculated as the proportion of all patients that received an antibiotic prescription post-intervention minus the same proportion measured in the pre-intervention period. For randomised controlled trials, pre-intervention differences in the proportion of prescriptions between treatment and control groups were subtracted from post-intervention differences. A negative effect size indicates that ASPs are associated with a reduction in antibiotic prescriptions of magnitude equal to the value of the effect size itself. Numbers quoted in percentage terms are the weights assigned to each effect size by the three-level random effects model. 95% confidence intervals are included in brackets.(Antimicrobial Stewardship program= multi-component ASPs)

© 2023 Zay Ya K et al. *JAMA Network Open.*

eFigure 3. Subgroup Analyses (Consumption in DDD per 100 Patient-Days)

A. Forest plot of included studies stratified by patient settings (Antibiotic consumption in DDD per 100 patient days)

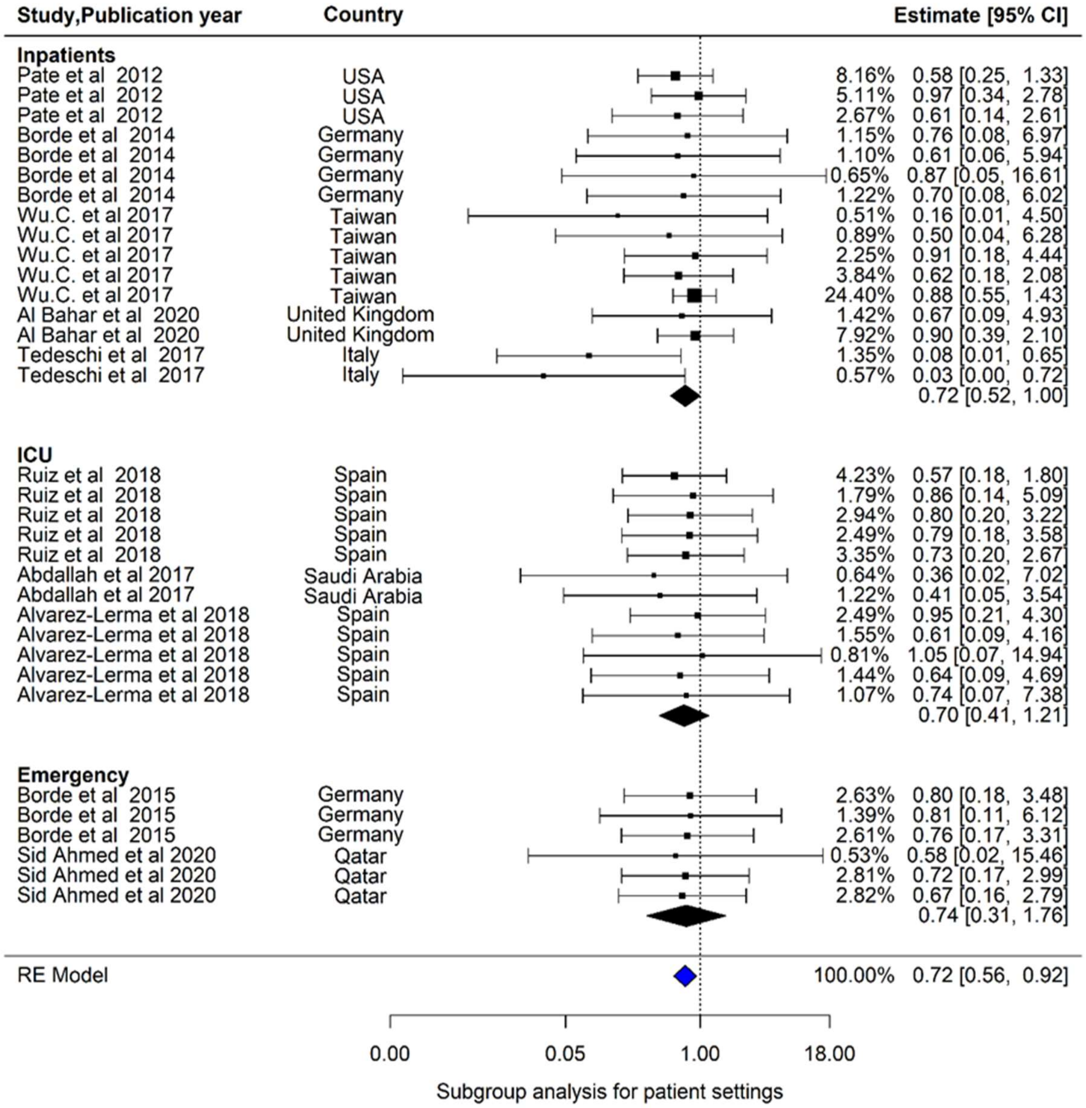

Figure 3a shows the stratified results for the average change in antibiotic consumption post- compared to pre-intervention. RR:rate ratio. The rate ratio (RR) of antibiotic consumption was obtained by dividing the post-intervention consumption rate measured in DDD per 100 PD by the pre-intervention consumption rate. A rate ratio below the value of 1 indicates that ASPs are associated with a reduction of (1-RR) % in antibiotic consumption. Numbers quoted in percentage terms are the weights assigned to each effect size by the three-level random effects model. 95% confidence intervals are included in brackets. (ICU= Patients in intensive care unit, Emergency = Patients in acute care hospital).

© 2023 Zay Ya K et al. *JAMA Network Open.*

B. Forest plot of included studies stratified by antibiotic restriction as per individual protocol settings (Antibiotic consumption in DDD per 100 patient days)

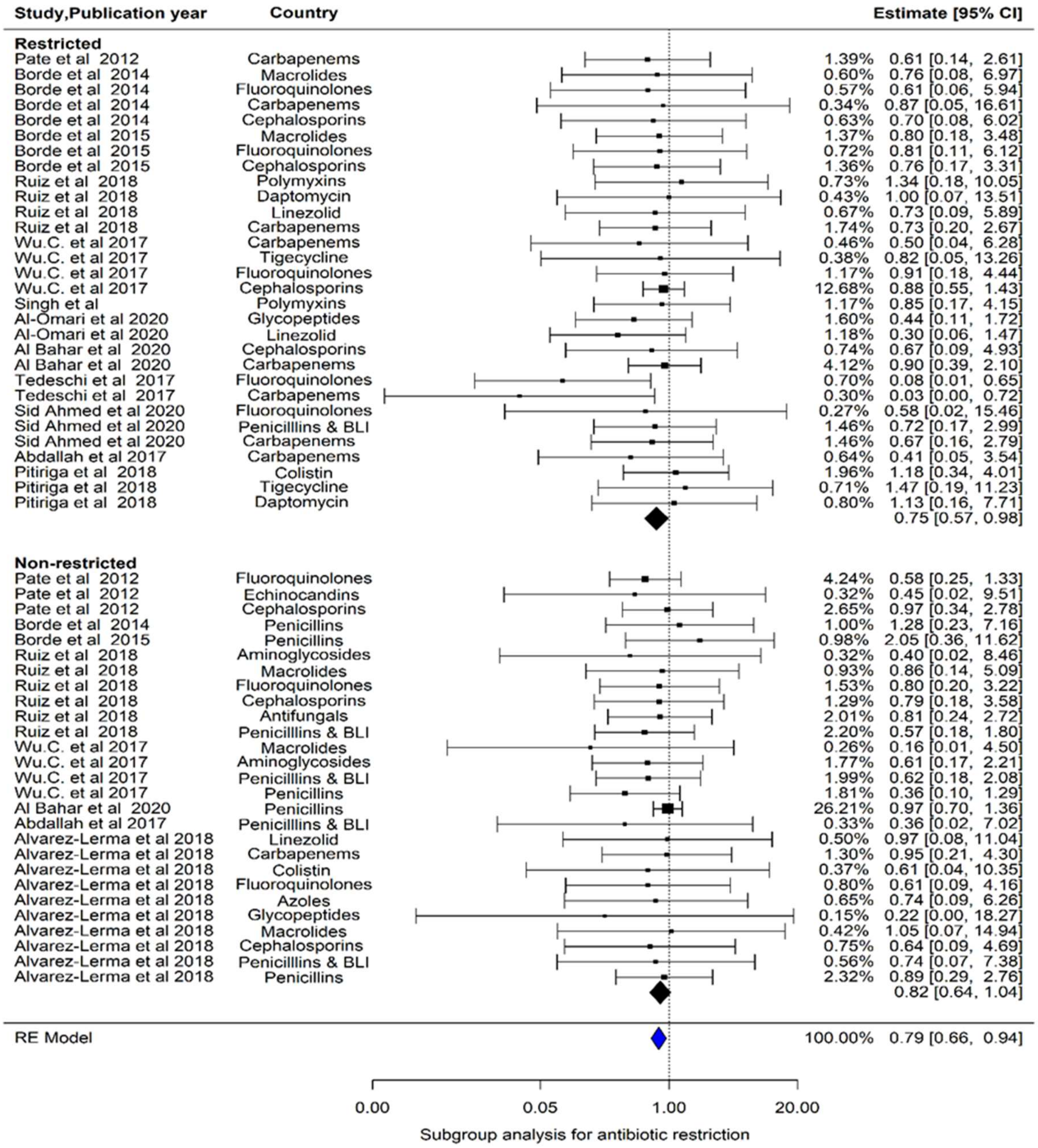

Figure 3b shows the stratified results for the average change in antibiotic consumption post- compared to pre-intervention. RR:rate ratio. The rate ratio (RR) of antibiotic consumption was obtained by dividing the post-intervention consumption rate measured in DDD per 100 PD by the pre-intervention consumption rate. A rate ratio below the value of 1 indicates that ASPs are associated with a reduction of (1-RR) % in antibiotic consumption. Numbers quoted in percentage terms are the weights assigned to each effect size by the three-level random effects model. 95% confidence intervals are included in brackets. Non-restricted= No restriction on antibiotic as per individual protocol).

© 2023 Zay Ya K et al. *JAMA Network Open.*

eFigure 4. Meta-analysis Summary (Antibiotic Consumption in DDD per 100 Patient-Days)

Panel A

Antibiotic classes	RR (95% CI)
Penicillins & BLI	0.61 (0.24,1.59)
Penicillins	0.94 (0.62,1.45)
Macrolides	0.74 (0.20,2.68)
Fluoroquinolones	0.58 (0.30,1.11)
Cephalosporins	0.85 (0.53,1.38)
Carbapenems	0.69 (0.38,1.25)

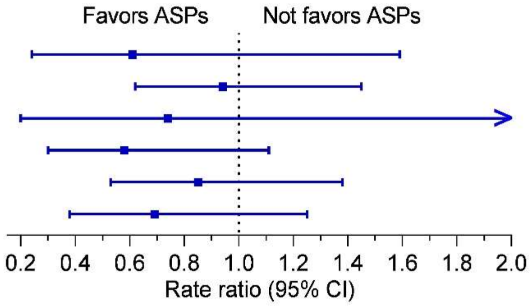

Panel B

AWaRe antibiotic classification	RR (95% CI)
Access	0.90 (0.65,1.25)
Watch	0.72 (0.56,0.92)
Reserve	0.77 (0.43,1.41)

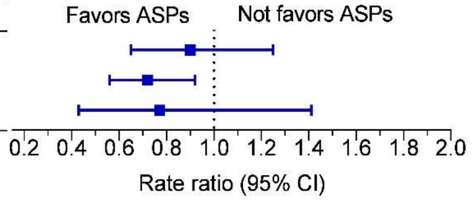

Panel C

Antibiotic restriction	RR (95% CI)
Restricted	0.75 (0.57,0.98)
Non-Restricted	0.82 (0.64,1.04)

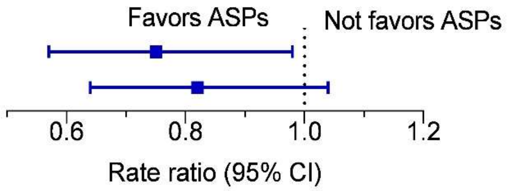

Panel D

Patient settings	RR (95% CI)
Inpatients	0.72 (0.52,1.00)
ICU patients	0.70 (0.41,1.21)
Emergency patients	0.74 (0.31,1.76)

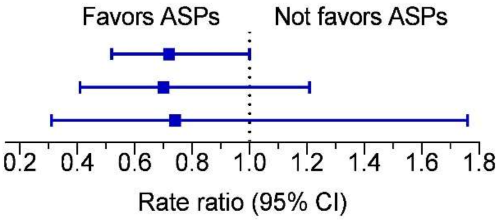

Figure 4 5 summarizes stratified results for the change in antibiotic consumption after ASPs in post- to the pre-intervention period. RR:rate ratio. The rate ratio (RR) of antibiotic consumption was obtained by dividing the post-intervention consumption rate measured in DDD per 100 PD by the pre-intervention consumption rate. A rate ratio below the value of 1 indicates that ASPs are associated with a reduction of (1-RR) % in antibiotic consumption. Error bars represents 95% CI and the size of each square represents the pooled effect size. Panel A: Stratified result by antibiotic classes, Panel B: Stratified result by AWaRe WHO antibiotic classification, Panel C: Stratified result by antibiotic restriction, Panel D: Stratified result by patient settings

© 2023 Zay Ya K et al. *JAMA Network Open.*
