# Supplementary material for: Association Between Antimicrobial Stewardship Programs and Antibiotic Use Globally: A Systematic Review and Meta-Analysis
Source: JAMA Netw Open. 2023 Feb 9;6(2):e2253806. doi: 10.1001/jamanetworkopen.2022.53806 (PMC9912134; doi:10.1001/jamanetworkopen.2022.53806)
Supplement: Supplement 2. — Data Sharing Statement [file jamanetwopen-e2253806-s002.pdf]

Data Sharing Statement

Zay Ya. Association Between Antimicrobial Stewardship Programs and Antibiotic Use Globally. *JAMA Netw Open*. Published February 09, 2023. doi:10.1001/jamanetworkopen.2022.53806

Data
Data available: Yes
Data types: Data dictionary, Other (please specify)
Additional Information: Supplementary materials
How to access data: All data and figures relevant to this systematic review and meta-analysis paper can be seen in the article or supplementary materials.
When available: With publication

Supporting Documents
Document types: None

Additional Information
Who can access the data: All data and figures relevant to this systematic review and meta-analysis paper can be seen in the article or supplementary materials.
Types of analyses: for any purpose
Mechanisms of data availability: without investigator support
